# Belowground Response to Drought in a Tropical Forest Soil. I. Changes in Microbial Functional Potential and Metabolism

**DOI:** 10.3389/fmicb.2016.00525

**Published:** 2016-04-20

**Authors:** Nicholas J. Bouskill, Tana E. Wood, Richard Baran, Zaw Ye, Benjamin P. Bowen, HsiaoChien Lim, Jizhong Zhou, Joy D. Van Nostrand, Peter Nico, Trent R. Northen, Whendee L. Silver, Eoin L. Brodie

**Affiliations:** ^1^Earth Sciences Division, Ecology Department, Lawrence Berkeley National LaboratoryBerkeley, CA, USA; ^2^International Institute of Tropical Forestry, USDA Forest ServiceRio Piedras, PR, USA; ^3^Fundación Puertorriqueña de ConservaciónSan Juan, PR, USA; ^4^Life Sciences Division, Lawrence Berkeley National LaboratoryBerkeley, CA, USA; ^5^Institute for Environmental Genomics and Department of Microbiology and Plant Biology, University of OklahomaNorman, OK, USA; ^6^State Key Joint Laboratory of Environment Simulation and Pollution Control, School of Environment, Tsinghua UniversityBeijing, China; ^7^Department of Environmental Science, Policy and Management, University of California-BerkeleyBerkeley, CA, USA

**Keywords:** tropical forests, drought, microbial ecology, osmolytes, functional gene microarray

## Abstract

Global climate models predict a future of increased severity of drought in many tropical forests. Soil microbes are central to the balance of these systems as sources or sinks of atmospheric carbon (C), yet how they respond metabolically to drought is not well-understood. We simulated drought in the typically aseasonal Luquillo Experimental Forest, Puerto Rico, by intercepting precipitation falling through the forest canopy. This approach reduced soil moisture by 13% and water potential by 0.14 MPa (from -0.2 to -0.34). Previous results from this experiment have demonstrated that the diversity and composition of these soil microbial communities are sensitive to even small changes in soil water. Here, we show prolonged drought significantly alters the functional potential of the community and provokes a clear osmotic stress response, including the production of compatible solutes that increase intracellular C demand. Subsequently, a microbial population emerges with a greater capacity for extracellular enzyme production targeting macromolecular carbon. Significantly, some of these drought-induced functional shifts in the soil microbiota are attenuated by prior exposure to a short-term drought suggesting that acclimation may occur despite a lack of longer-term drought history.

## Introduction

A fundamental goal of microbial ecology is to understand the relationships between microbial community composition and ecosystem function. The fitness of endemic microorganisms is dictated by a suite of traits (Green et al., 2008; Allison, 2012), the combination of which are selected for through historical pressures such as climate, resource availability, and resource stoichiometry (David and Alm, 2010). While the time scales are not well-defined, it is assumed that microorganisms inhabiting a specific environment bear the hallmark of localized integrated selective pressures (e.g., Gianoulis et al., 2009; Coleman and Chisholm, 2010). This selection and structuring by antecedent conditions is especially relevant in the face of new climate regimes, where ecosystems may experience conditions beyond existing climate variability (Bonebrake and Mastrandrea, 2010). For this reason, the biotic responses to a changing climate may largely be determined by the adaptive capacity of the communities undergoing change and the rapidity with which that change occurs (Deutsch et al., 2008).

Humid tropical forests are globally important carbon reservoirs (Jobbágy and Jackson, 2000; Pan et al., 2011), maintained, in part, by a relatively aseasonal warm climate with little variability in the magnitude or frequency of throughfall (i.e., rainfall not intercepted by the canopy). Earth system models predict large-scale changes to precipitation patterns in the tropics with overall declines in precipitation expected in the subtropics and dry tropics and increases in precipitation in the wet tropics. Models further predict that wet-dry seasonality in the tropics will increase (Collins et al., 2013; Fu et al., 2013). While it remains unclear whether droughts will increase in duration or intensity (Collins et al., 2013; Kirtman et al., 2013), as a whole, the temporal variation of precipitation is likely to change. Humid tropical forests are an ideal ecosystem to examine the impact of rarely experienced perturbations (e.g., drought) on a microbial community with an adaptive capacity shaped by a relatively aseasonal climate.

Recent work in humid tropical forests has demonstrated that microbial communities may be acclimated to rapid changes in redox or oxygen (O_2_) concentrations (Pett-Ridge and Firestone, 2005; DeAngelis et al., 2010), but this acclimation is unlikely to extend to changes in water potential that are rarely experienced in this ecosystem (Ψ, Bouskill et al., 2013). Water availability is among the most important controls on soil biological activity (Manzoni et al., 2012), and small changes to soil moisture can lead to large changes in the structure of microbial communities (Carney and Matson, 2006; Bouskill et al., 2013; Evans et al., 2014; Waring and Hawkes, 2015), albeit with a degree of phylogenetic conservatism (Schimel et al., 2007) conforming to presumptive ecological strategies (Evans and Wallenstein, 2014). The impact of a decline in soil water potential manifests as a set of interrelated physical and biological impacts (Stark and Firestone, 1995; Or et al., 2007; Schimel et al., 2007). Physically, decreased soil water content may constrain the diffusion of substrates or extracellular enzymes, thereby slowing biogeochemical rates and limiting decomposition due to associated substrate limitation (Stark and Firestone, 1995; Allison, 2005). At a physiological level, drought may result in a resource allocation shift (Schimel et al., 2007) to facilitate the uptake or synthesis of osmolytes and compatible solutes for osmotic balance (Potts, 1994; Welsh, 2000) or spore formation (Stark and Firestone, 1995).

The current study builds on recent work examining the phylogenetic response of humid tropical forest soil microbes to changing water availability. This previous study demonstrated that a 10 months drought, imposed through the installation of rainfall shelters, significantly reduced the phylogenetic richness and composition of forest soil microbial communities in treatment soils exposed to drought for the first time (a treatment hereafter referred to as “*de novo*”), despite no change in total biomass (Bouskill et al., 2013). However, a parallel experiment in treatment soils that had previously undergone short-term (3 months) experimental drought (hereafter, referred to as “pre-excluded”) 1 year prior to the 10 months drought experiment described here, provided evidence that microbial communities within humid tropical forests may rapidly acclimate to repeat perturbation.

Here, we use genomic and metabolomics (osmolyte) measurements and enzyme activity assays to develop an understanding of whether these phylogenetic changes alter the functional ecology and activity of these soil communities. Despite a theoretical link between the phylogenetic and functional potential of microbial communities, there are few studies that have definitively examined these relationships. It is possible that microbial communities contain sufficient functional redundancy to negate the effect of phylogenetic change (Allison and Martiny, 2008). However, the distributions of certain functions (e.g., complex-C hydrolyzing enzymes) are non-random and, in some cases, overrepresented within specific taxonomic groups (Berlemont and Martiny, 2013; Zimmerman et al., 2013). Therefore, changes to the diversity and abundance of phylogenetic taxa with functional capabilities unique to, or enriched in, their specific clade has the potential to alter subsurface biogeochemistry (Allison et al., 2013). Herein, we compare the drought treatments to control soils and ask: (1) Does drought induce a significant change in the microbial function in soils with low antecedent variability in soil moisture? (2) Does a metabolic stress response (e.g., osmolyte production) play an important role in a tropical soil with little history of fluctuating Ψ? (3) How does pre-exposure to drought affect the adaptive capacity and microbial functional potential under repeat exposure?

## Materials and Methods

### Throughfall Exclusion

This study was conducted in a humid tropical forest in the Bisley Research Watershed of the Luquillo Experimental Forest (LEF) in Puerto Rico (~350 m a.s.l; 18° 18 N, -65° 50 W). A detailed classification of these soils has been published previously (Silver et al., 1994). Briefly, these soils are classified as ultisols in the Humatus-Cristal-Zarzal series, and are derived from volcanic sediments with Tertiary-age quartz-diorite intrusions of the Rio Blanco stock. The soils are deep, clay rich and acidic, with high aluminum and iron content (Scatena, 1989). The throughfall exclusion experiment was established in a Tabonucco forest stand located on an upper ridge in June 2008 with an initial 10 soil plots (Wood and Silver, 2012). A description of the experiment has also been published previously (Bouskill et al., 2013). Briefly, throughfall was excluded from five of the plots with clear, corrugated plastic panels (1.54 m^2^) mounted 1 m above the forest floor at a 17° angle, for a period of 3 months before the shelters were removed and ambient throughfall resumed (“pre-excluded”). The five remaining plots were not sheltered and served as controls. The plots were not trenched to minimize soil disturbance and allow lateral movement of water across the plots, which focuses our experiments on the affect of reducing throughfall. The following year (June, 2009), the shelters were replaced over these original five throughfall-excluded plots and five new (“*de novo”*) exclusion experiments were established (15 plots total). Soil samples (5–10 g) were taken in triplicate from each soil plot after 3 months of throughfall exclusion and again 10 months following the placement of throughfall shelters. In the present work we primarily focus on the samples collected after 10 months treatment. Soil samples were shipped overnight to Berkeley at 4°C.

The high soil clay content did not allow for direct extraction of porewater. Therefore, soil water was extracted from 15 g of fresh soil by adding 15 ml ultra-pure water and vortexing (10 min). Samples were filtered through a 0.2 μm nylon filter for 30 min at 2,500 rpm at 4°C. The extracted soil water was used to measure pH, conductivity, cation and anion concentrations, water potential, and total organic carbon (TOC), according to (Bouskill et al., 2013). The final volume of extracted soil water, minus the additional 15 ml, was taken as the soil water and measured cations, anions, and carbon concentrations normalized to this volume and expressed in mM (ions) or mg L^-1^ (TOC). Fresh samples were used for enzyme assays. Soil moisture was measured by drying 5 g of soil at 105°C over 24 h, with the difference between the initial and final weights taken as the water content. The remaining soil was frozen at -80°C until nucleic acid extraction.

### GeoChip Analysis of Functional Potential

Target amplification, labeling and hybridization protocols have been described previously (Lu et al., 2011). Briefly, total nucleic acids were extracted from 0.5 g of soil per replicate according to the cetyl trimethyl ammonium bromide/bead-beating-based protocol described previously (Ivanov et al., 2009). DNA was Cy-3 labeled and hybridized to the GeoChip 4.0 (synthesized by NimbleGen, Madison, WI, USA) at 40°C with 40% formamide for 16 h on a Maui hybridization station (BioMicro, Salt Lake City, UT, USA). GeoChip 4.0 contains 82,000 probes that cover over 141,995 coding sequences representing 410 functional gene families related to microbial carbon, and nutrient cycling, energy metabolism, antibiotic resistance, metal resistance/reduction, organic remediation, stress responses, bacteriophage, and virulence. Following hybridization, arrays were scanned (NimbleGen MS200, Madison, WI, USA), signal intensities measured, and spots with signal to noise ratio < 2 removed. Data analysis was concentrated on genes within four categories related to C and nutrient cycling [C degradation and nitrogen (N)/phosphate (P) cycling genes], and genes related to stress pathways.

### Beta Diversity Analysis

Raw geochip data was manually edited to remove poorly replicated features. The data was subsequently log transformed and converted into a weighted distance matrix using chi-squared distance, calculated using the R package labdsv (Roberts, 2007). We examined how drought affected functional potential using, (1) community ordinations to project the spatial dissimilarity between control and treatment soils, (2) variance partitioning methods (multi-response permutation procedure and permutation multivariate anova) to determine the proportion of observed changes in the functional potential relatable to physico-chemical factors, and, (3) canonical correspondence analysis (CCA) to linearly correlate environmental variables with biological variables and link the functional potential with an array of environmental variables. All analyses were performed using the vegan package in R (Oksanen et al., 2013).

### Soil Enzyme Assays

The potential activities of several enzymes involved in soil C cycling were quantified following prolonged (10 months) drought treatment from fresh soil material within 24 h of collection according to (DeAngelis et al., 2011). Enzyme assays targeted the hydrolytic enzymes (alongside the individual methylumbelliferyl-labeled substrates in italics): β-1,4-glucosidase (BG; β-D*-glucopyranoside*), catalyzing the degradation of cellobiose to glucose; Cellobiohydrolases (CBH; β-D*-cellobioside*), a group of extracellular-cellulases responsible for the hydrolysis of cellulose to cellobiose; *N*-acetyl-D-glucosaminidase (NAG; *N-acetyl-*β-D*-glucosaminide*), which hydrolyses chitin and peptidoglycan; Xylanase (Xyl; β-D*-xylopyranoside*), which hydrolyses linear polysaccharides to xylose. Substrates (Sigma-Aldrich) for each enzyme were dissolved in 0.05 M sodium acetate buffer (pH 5.5) to a 10 mM concentration. Soil samples were weighed and homogenized slurries created by vigorously stirring 1 g of soil in 50 ml 0.05 M sodium acetate buffer. Sub-samples of the slurries were removed to 96 well plates, incubated with 50 μl of the relevant substrate (1X concentration) and immediately quantified by fluorimetry (excitation = 365 nm; emission = 442 nm). Assay plates were incubated at 27°C and fluorescence quantified after 4 and 24 h following substrate addition. Final enzyme activities are expressed as nmol g soil^-1^ h^-1^. We tested the effects of experimental drought on enzyme activity by performing two-way analysis of variance (ANOVA) comparing the control with treatments.

### Osmolyte Separation

To examine potential biological responses to changing water potential we examined the abundance of several osmolytes in the unfiltered soil water following 10 months of drought. The vigorous nature of the extraction method likely disrupts and lyses cell walls effectively extracting both intracellular and extruded metabolites. Polar compounds were extracted by adding 1 ml methanol (-20°C) to the solution, vortexing for 30 s and incubating at -20°C for 3 min. Following incubation the solution was centrifuged for 1 min at 2,350 *g* and 750 μl removed to a glass vial. Non-polar metabolites were extracted by adding 500 μl hot isopropanol (65°C) to the solution and incubating at 65°C for 3 min. The solution was centrifuged for 14,000 *g* and 750 μl removed to a glass vial. Both polar and non-polar supernatants were concentrated by spinvac and redissolved in 100 μl of methanol containing an internal standard, 1 μg ml^-1^ of 2-amino-3-bromo-5-methylbenzoic acid. The samples were stored at 4°C, filtered through a 0.2 μm PVDF membrane microcentrifugal filter (National Scientific) and analyzed via LC–MS using normal phase liquid chromatography (ZIC-HILIC capillary column, Agilent 1200 series capillary LC system) coupled to a quadrapole time-of-flight mass spectrometer (Agilent 6520 dual-ESI-Q-TOF). Run in positive and negative mode, this method gave signal intensity and spectra data across a wide range (m/z range 52.08–1663.03) from each of the 15 samples. From the raw data, MassHunter software (Agilent, Santa Clara, CA, USA) was used to define and quantify peaks representing different molecules (e.g., trehalose, ectoine), and standards were used to confirm targeted metabolites of interest.

## Results

### Soil Physicochemical Response

A detailed summary of the effect of experimental drought on the chemistry of these soils has been published previously (Bouskill et al., 2013). Drought reduced soil moisture and altered soil water chemistry (Supplementary Table S1). Prolonged (10-months) drought resulted in lower soil moisture values for the pre-excluded (~65% soil moisture) and *de novo* soils (~60%) compared to the control (~76%, Supplementary Table S1). As previously reported (Bouskill et al., 2013), after the 10-months drought phosphorus (P) and several redox sensitive compounds, e.g., aluminum (Al), iron (Fe) and molybdenum (Mo), were significantly lower in soil water extracts (Supplementary Table S1). Soil Ψ was also significantly lower in soils undergoing drought (pre-excluded = -0.27 ± 0.06; *de novo* = -0.34 ± 0.1 MPa) than the control soils (-0.19 ± 0.03 MPa), while sodium (Na) and potassium (K) concentrations in soil water were significantly higher (*p* = 0.01).

### Response of Soil Microbial Community Functional Potential

**FIGURE 1:**
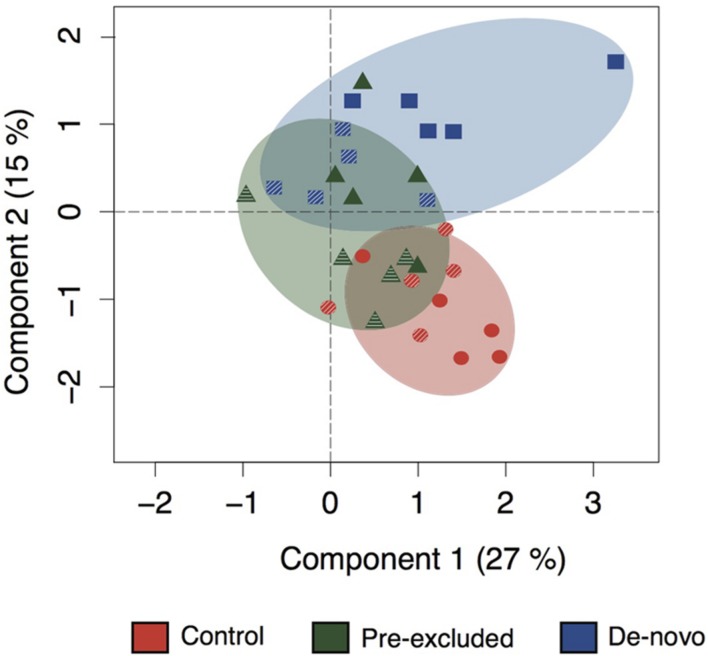
**Ordination showing changing functional potential across the experimental duration.** The soils are color-coordinated as either control (red), pre-excluded (green), and *de novo* soils (blue). The hashed symbols indicate the 3 months sampling point, and the filled symbols represent the 10 months sampling point. The statistical dissimilarity for this ordination can be found in Supplementary Table S2.

Using functional gene array hybridization we examined the functional potential (i.e., the complement of functional genes within a community) of control and drought soils. Ordination plots of the complete suite of functional genes represented on the Geochip showed that short-term (3-months) drought had a larger impact on the functional potential of the *de novo* excluded soil microbial communities relative to those in control or pre-excluded soils (**Figure 1**, Supplementary Table S2). The *de novo* soils diverged further from controls relative to the pre-excluded soils along both ordination axes. As drought continued (to 10-months), the *de novo* soil microbial functional potential became significantly different from the control soils and the pre-excluded treatments (**Figure 1**, Supplementary Figure S1, Supplementary Table S2). Pre-excluded soils at 10 months also became distinct from the control soils, with a functional potential more similar to that of the *de novo* soils following short-term (3-months) drought.

**FIGURE 2:**
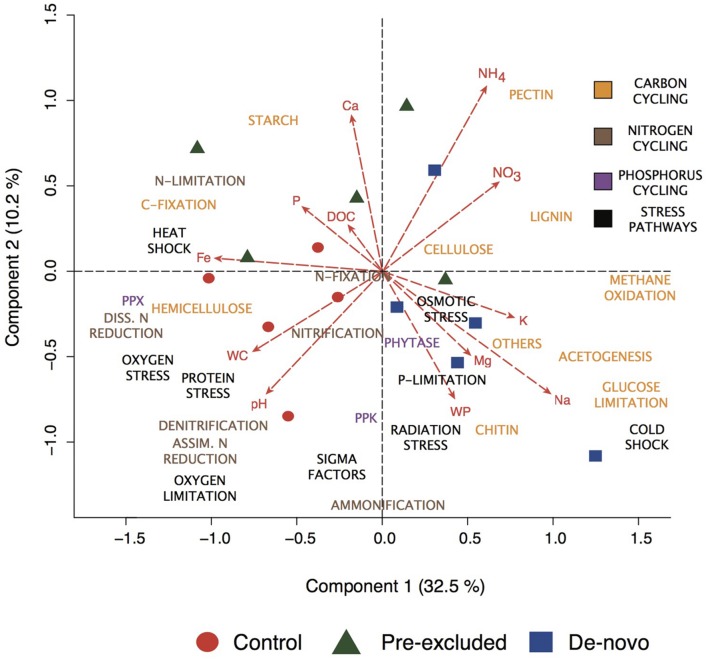
**Canonical correspondence analysis (CCA) of the functional potential of the soils as indicated by genomic analysis following 10 months of throughfall exclusion.** Data shown are restricted to four functional gene categories (carbon degradation, nitrogen and phosphorus cycling, and stress pathways), and the major functional groups within each these categories are ordinated onto the plot and grouped by color (as denoted by the inset in the upper right corner). The soils are also color-coordinated as either control (red), pre-excluded (green), and *de novo* soils (blue). The functional potential of the control and treatment soils are correlated against the collected physicochemical factors and the relationship projected onto the ordination. The directionality and length of the vectors is proportional to the direction and length of the chemical gradient sampled from the soil water. Vector identity is indicated by the abbreviated label at the tip of the arrow (NH_4_, ammonia; NO_3_, nitrate; K, potassium; Na, sodium; Mg, magnesium; WP, water potential; WC, water content; Fe, iron; P, phosphate; DOC, dissolved organic carbon; Ca, calcium).

We subsequently focused our analyses on the response of a relevant subset of genes related to carbon degradation/transformation, nitrogen and phosphorus cycling, and physiological stress that explained 42% of the overall variance in functional potential. Prolonged drought (10 months) clearly shaped the functional potential (**Figure 2**), with the pre-excluded and *de novo* treatment soils becoming more similar to each other relative to the control soils for each gene category (Supplementary Figures S1B–E). CCA showed the separation of control and experimental soils could be explained by the differential distribution of specific gene categories (**Figure 2** and Supplementary Figure S2). This included a higher relative abundance of genes involved in complex C-degradation (chitin, cellulose, and lignin), nutrient limitation and osmotic stress response in the *de novo* excluded soils relative to the controls. Genes involved in C-degradation correlated negatively with soil water content, but positively with nitrogen species. Genes related to P limitation were enriched in the *de novo* soils, and negatively correlated to soil water P concentration. Control soils had a higher relative abundance of genes related to O_2_ limitation and anaerobic processes [e.g., denitrification, dissimilatory nitrate (NO_3_) reduction; **Figure 2** and Supplementary Figure S2]. The prevalence of these genes is likely related to the higher reduction potential of these soils, inferred from higher soluble Fe concentrations relative to the soils undergoing drought (Liptzin and Silver, 2009; Dubinsky et al., 2010; Bouskill et al., 2013). At a categorical level, genes related to hemicellulose decomposition ordinated alongside the control samples (**Figure 2**) implying a higher relative abundance compared to drought treatments. However, at finer resolution (i.e., individual genes coding for hydrolysis of specific hemi-cellulose polysaccharides) genes related to arabinan breakdown were higher within the control soils, while those related to xylan hydrolysis were enriched in drought soils (Supplementary Figure S3).

The relationship between the functional potential and edaphic properties was also analyzed by permutational non-parametric multivariate analysis of variance (perMANOVA) considering the following variables: (1) treatment (control or drought); and (2) Ψ, Na, P, and Fe concentration and the interaction between these variables (Supplementary Table S3). Following 3 months of treatment we discerned no significant relationships between the functional potential of the soils and the different treatment variables (Supplementary Table S3A). However, the functional potential of the soil microbial community 10 months following the placement of throughfall shelters was significantly correlated with Na (*R*^2^ = 0.11; *p* = 0.02). Genes involved in C, N, and P cycling and physiological stress responses were significantly related to the interaction of Na-P (Supplementary Table S3B). Phosphorus concentrations explained 11% of the variance in N and P-cycling genes (Supplementary Table S3B).

### Response of Soil Extracellular Enzyme Activity

**FIGURE 3:**
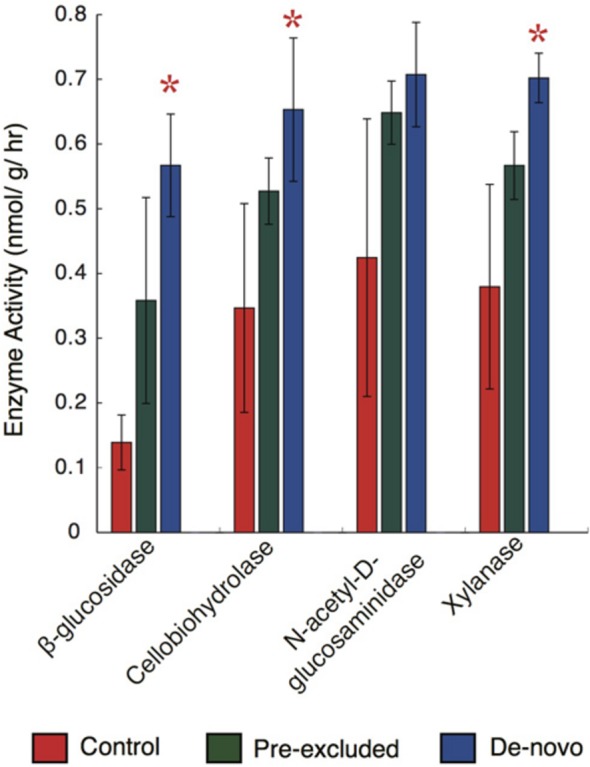
**Hydrolytic enzyme activity across the control and treatment soil plots following 10 months of throughfall.** The bar plots represent the mean (± standard deviation) of activities across the treatments (*n* = 5). Stars above the plots denote significant differences when compared with the control.

We subsequently asked whether significant changes in the functional potential of the soil microbial communities would be expressed as alterations in extracellular enzyme activity. We focused on the potential activity of enzymes related to depolymerization of plant and microbial macromolecules (BG, CBH, Xyl, NAG) following 10 months of drought. With the exception of NAG, enzyme activity was significantly higher in the *de novo* treatments relative to the control soils (two-way ANOVA, *p* < 0.05; **Figure 3**). Mean soil enzyme activities for BG, CBH, and Xyl were 40, 30, and 46% higher than the controls, respectively. Enzyme potential in the pre-excluded soils was generally elevated above the controls soils but lower than the *de novo* soils. No significant difference was noted between the pre-excluded and control soils.

### Osmolyte Response

**FIGURE 4:**
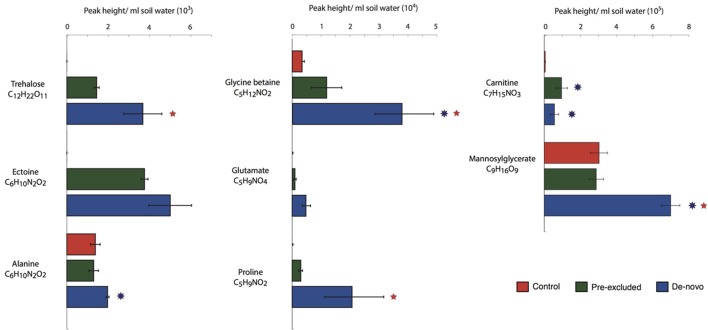
**Abundance of metabolites positively identified as compatible solutes following 10 months of throughfall exclusion.** The bar plots represent the mean (± standard deviation) of activities across the treatments (*n* = 5). The symbols next to the bars denote significantly different between one of the treatments and control (red star) soils or significant different between treatment soils (blue star).

Using an untargeted LC–MS/MS approach (environmental metabolomics) we identified a number of compatible solutes that are either synthesized or accumulated by microbial cells under osmotic stress. Both trehalose and ectoine were detected solely in soils undergoing drought (**Figure 4**). Trehalose abundance was also significantly elevated in the *de novo* excluded soils relative to the pre-excluded (*p* < 0.05). Glycine betaine was significantly higher in the *de novo* excluded soils relative to either the control or pre-excluded soils (*p* < 0.001). Glycine betaine was also in higher abundance in the pre-excluded soils relative to the controls. Several amino acids (e.g., alanine, glutamate, proline), that have been shown to accumulate during osmotic stress, were in higher relative abundance in the *de novo* soils than either the pre-excluded or the control soils (**Figure 4**). Mannosylglycerate was significantly higher in the *de novo* soils relative to the control and pre-excluded (*p* < 0.001). Finally, carnitine was significantly higher in both of the treatment soils relative to the control (*p* < 0.05).

## Discussion

### Microbial Response to Prolonged Drought

Prolonged drought in these humid tropical forest soils resulted in a significant change in the distribution of microbial functional genes, reflecting the previously reported large shifts in phylogenic diversity and community structure toward increasing representation of Actinobacteria and Planctomycetes (Bouskill et al., 2013). Furthermore, the observed shifts occurred *in lieu* of any change in microbial biomass (Bouskill et al., 2013), signifying that the switch in the functional potential of the treatment plots originate from changing community composition. Genes related to osmotic stress response pathways (e.g., osmotically inducible proline and betaine transporters) were enriched within both the pre-excluded and *de novo* soils relative to the controls suggesting selection for organisms with these traits. Environmental metabolomic data demonstrated that osmotic stress occurred despite modest changes in soil water potential, with elevated concentrations of compatible solutes (including glycine betaine and trehalose) observed in soils undergoing drought (**Figure 4**). This implies that the ability to adapt to water potential stress creates a selective pressure partially responsible for a previously reported shift in community composition (Bouskill et al., 2013). The synthesis and accumulation of osmolytes is a well-documented response of bacteria to osmotic stress (Welsh et al., 1991; Potts, 1994; Welsh, 2000), attributed to a need to maintain cellular turgor and preserve intracellular macromolecular structure (Csonka, 1989; Kempf and Bremer, 1998).

Evidence for the production of osmolytes under drought is ambiguous, with studies focus on microbial response *in situ* measuring a significant increase in osmolyte production under soil drying (Warren, 2014) or no effect (Boot et al., 2013; Kakumanu et al., 2013). This reported difference can be attributable to the rate and extent of soil drying, the resource availability to support a metabolic response to drought, or the frequency of drought shaping the adaptive capacity to future perturbation (i.e., life history traits associated with a community, Lennon et al., 2012; Manzoni et al., 2014).

Theoretical studies have shown that the large carbon investment required to synthesize osmolytes is uneconomical under very dry conditions (Manzoni et al., 2014). At low Ψ (i.e., -14 MPa) water films shrink and become disconnected, constraining substrate diffusivity (Moldrup et al., 2001; Rodriguez-Iturbe and Porporato, 2005), microbial activity (Manzoni et al., 2012) and cellular dispersal (Dechesne et al., 2010). These conditions are commonly found within Mediterranean grassland soils (Boot et al., 2013), where Ψ declines significantly during seasonal drought (< -20 MPa, Kieft et al., 1987; Placella et al., 2012). Therefore, under very dry conditions, the large carbon investment required to synthesize osmolytes creates an intracellular carbon demand that is likely unfulfilled by corresponding substrate diffusion rates. Moreover, because the monomer return from producing extracellular enzymes is low under these conditions, an investment/tolerance strategy against drought is likely selected against (Manzoni et al., 2014). Conversely, rapid phenotypic switching of the microbial community to a dormant state could maintain cellular viability from which microbes resuscitate under resumption of precipitation (Bar et al., 2002; Placella et al., 2012; Rajeev et al., 2013).

Here, however, drought induced changes in soil Ψ and soil moisture were modest (Supplementary Table S1), and unlikely to constrain substrate, nutrient, or enzyme diffusion in soil or impair microbial physiological function (Rodriguez-Iturbe and Porporato, 2005; Manzoni et al., 2012; Manzoni and Katul, 2014). Nevertheless, soil solute concentrations (e.g., NaCl) played a role in driving the discrimination between control and treatments, while a clear osmotic response was mounted to counteract the elevated solute concentrations in the treatment plots suggesting these microorganisms are sensitive to modest changes in Ψ. Relative to drier soils, the production and accumulation of osmolytes may be a favorable ecological strategy (Manzoni et al., 2014), selecting for distinct taxonomic groups (Bouskill et al., 2013).

Changes to the microbial community functional potential under drought occurred concomitant with an increase in the activity of hydrolytic enzymes responsible for the breakdown of complex organic C. The functional gene data found genes encoding extracellular-enzymes that degrade chitin, cellulose, lignin, and pectin, and enzymes involved in hemicellulose (xylose) catabolism (Supplementary Figure S2B), were of higher relative abundance in soils undergoing drought. The specific activities of the corresponding classes of enzymes were also higher in soils undergoing drought (**Figure 3**). Moreover, in a contemporaneous study (Bouskill, companion paper), we measured the concentrations of chemically complex organic compounds targeted by these enzymes and found them to be of lower concentration under treatment relative to control soils.

This demonstrates that within these soils measured changes in microbial functional potential are linked to changes in activity and the properties of soil carbon. Previous studies, however, have not found such an explicit link between drought and enzyme activity (Sardans and Penuelas, 2005; Bell et al., 2009; Steinweg et al., 2012). For example, Alster et al. (2013) showed a variable response of enzyme activity to soil moisture dependent on enzyme class, where hydrolytic enzyme activity generally increased under drought, and oxidative enzyme activity declined or did not change.

Increased potential enzyme activity can arise from soil oxygenation stimulating community metabolism (Allison, 2005; Fenner and Freeman, 2011), or via changes in community composition (Bouskill et al., 2013), such as the increased relative abundance of desiccation tolerant Actinobacteria (Davet, 2004; Okoro et al., 2008) producing complex-carbon degrading enzymes. Abiotic factors reducing the efficacy of extracellular-enzyme activity and/or an increased carbon demand due to the microbial metabolic response to drought might also play a role. We briefly elaborate on these latter two scenarios below.

The small decline in soil moisture is unlikely to significantly hinder substrate diffusion, at least at the microscale (Manzoni and Katul, 2014), yet enzyme activity or substrate availability may still be attenuated through an increasing sorptive capacity of soil minerals (Allison, 2006). Changes to redox cycling in soils undergoing drought can alter the surface area of Fe-rich soil minerals (e.g., goethite), that play an important role determining the availability of soil C and specific activity of extracellular enzymes (Torn et al., 1997; Thompson et al., 2006; Schmidt et al., 2011; Vogel et al., 2014). Enzyme sorption to mineral surfaces can stabilize or enhance activity (Tietjen and Wetzel, 2003; Allison and Jastrow, 2006), albeit with exceptions (Naidja et al., 2000) that likely dependent on mineral-enzyme coupling (Allison, 2006). Therefore, under drought, the measured increase in enzyme activity might plausibly be attributed to either the prolonged reactivity of mineral associated enzymes (Kandeler, 1990), or due to intracellular carbon allocation toward enzyme production for the acquisition of carbon from sequestered by soil minerals.

Alternatively, while the synthesis of compatible solutes is a metabolically costly strategy (Potts, 1994; Manzoni et al., 2014), it can provide drought-tolerant organisms with a competitive advantage during periods of fluctuating osmotic potential. However, given a likely increase in intracellular carbon demand (Schimel et al., 2007), the viability of this strategy is dependent on substrate availability (Schimel et al., 1989; Manzoni et al., 2014). In this case, the consumption of available carbon could stimulate the production of extracellular enzymes (Allison and Vitousek, 2005) to fulfill the cellular demand. The trade-off in the metabolic tolerance of osmotic stress would likely reduce the fitness of an organism in the absence of stress (Gudelj et al., 2010; Evans and Wallenstein, 2014), partially explaining the shifts in community composition we observe under drought (Bouskill et al., 2013). To our knowledge there is little evidence from previous studies of microbial communities in tropical forest soils to support or refute our assertion. However, given the importance of microbial communities in belowground biogeochemical cycling (Bardgett et al., 2008; Bardgett and van der Putten, 2015), this work underlines the importance of further characterizing the response of microbial communities to changing precipitation patterns and increased drought frequency (Dai, 2012). In particular, it raises the pertinent question of whether microbial communities in tropical forest soils can rapidly adapt to repeated perturbation.

### Comparison of *De Novo* and Pre-excluded Functional Response under Drought

Our previous work has shown that a 3-months drought 1 year prior to commencement of the present study conditioned the pre-excluded soil microbial community to resist repeat drought relative to the *de novo* soils (Bouskill et al., 2013). Evidence for the rapid adaptation of microbial communities to repeat perturbation is emerging (Bowen et al., 2011; Evans and Wallenstein, 2011), however, little is known of the functional consequences of this conditioning. In the present study, the *de novo* soils show distinct changes in functional potential after 3 months of drought while the pre-excluded soils respond more slowly, initially resisting repeat perturbation. Prolonged drought (i.e., following 10 months) alters the functional potential of the pre-excluded soils toward a community structure similar to the *de novo* soils collected 3-months into the experiment but distinct from the *de novo* community that emerges after 10 months drought (**Figure 1**). This contrasts with the overlapping phylogenetic composition of pre-excluded and *de novo* soils following prolonged drought (Bouskill et al., 2013), and likely reflects the variability in functional potential not encompassed by phylogeny.

Several previous studies, focusing on either macro- or microorganisms, have demonstrated that pre-conditioning to perturbation increases the predictability of community succession (Chase, 2007; Lazzaro et al., 2011; Pagaling et al., 2013). While this predictability may demonstrate that ecological drift and priority effects are overridden by environmental filtering of tolerant organisms and selection for dry-adapted taxa (Chase, 2007), there is also likely a strong physical component influencing our results (see Bouskill et al., companion manuscript).

In the present work, stabilization of the pre-excluded soils undergoing repeated perturbation was evident through a more moderate response. Compared to the *de novo* soils the pre-exposed soils showed less divergence in the functional potential relative to the control soils, a lower osmolyte concentration and lower enzyme response. Furthermore, we have previously shown the phylogenetic response of the pre-excluded soils to be more constrained than that of the *de novo* soils (Bouskill et al., 2013). All of which points to the capacity of these tropical forest soil communities to develop a degree of resistance to repeat perturbation, possibly through physiological acclimation (DeAngelis et al., 2010; Bradford, 2013), a reduction in lag phase under repeat perturbation (Lambert and Kussel, 2014), or a hysteretic persistence of gene expression for specific responses (Mitchell et al., 2009; Lambert and Kussel, 2014).

## Conclusion

We have demonstrated here that a modest change in water potential (from -0.2 to -0.34) may fundamentally alter the functional potential and activity of tropical soils in Puerto Rico, albeit with clear potential for communities to adapt. However, while the genomic, metabolomic, and enzyme activity data are metrics of modified microbial activity, it is not unequivocal evidence of a changing carbon cycle. Given the central role microorganisms play in biogeochemical cycling (Singh et al., 2010; Bardgett and van der Putten, 2015) a logical follow-up question would focus on whether the composition and structure of the dissolved and bulk carbon pools are significantly altered by changing microbial functional potential and what role perturbation plays in the sensitivity or stability of these globally significant carbon sinks (Pan et al., 2011).

## Author Contributions

NB performed the research, analyzed the results. ZY, HL, RB, BB, JVN, JZ contributed to data collection and analysis. TW and WS, established the throughfall treatments in Puerto Rico. PN provided consultation for the work. TN provided novel methodologies. EB, TW, and WS designed the research. NB and EB wrote the manuscript, with contribution from all co-authors.

## Conflict of Interest Statement

The authors declare that the research was conducted in the absence of any commercial or financial relationships that could be construed as a potential conflict of interest.
