# Supplementary material for: Belowground Response to Drought in a Tropical Forest Soil. I. Changes in Microbial Functional Potential and Metabolism
Source: Front Microbiol. 2016 Apr 20;7:525. doi: 10.3389/fmicb.2016.00525 (PMC4837414; doi:10.3389/fmicb.2016.00525)
Supplement: Supplementary file 1 [file Data_Sheet_1.DOCX]

**Supplemental Material**

**Belowground response to drought in a weathered tropical forest soil. I. Functional changes in microbial composition are induced by decreasing water potential**

Nicholas J Bouskill^a*^, Tana E Wood^b,c^, Richard Baran^d^, Zaw Ye^a^, Ben P Bowen^d^, HsiaoChien Lim^a^, Jizhong Zhou^a,e,f^, Joy D Van Nostrand^e^, Peter Nico^a^, Trent R Northen^d^, Whendee L Silver^g^, & Eoin L Brodie^a,g*^

^a^Climate and Ecoystems Sciences Division, Lawrence Berkeley National Laboratory, Berkeley, CA, 94720

^b^International Institute of Tropical Forestry, USDA Forest Service, Rio Piedras, PR, 00926.

^c^Fundación Puertorriqueña de Conservación, San Juan, PR, 00936

^d^Environmental Genomics and Systems Biology Division, Lawrence Berkeley National Laboratory, Berkeley, CA, 94720.

^e^Institute for Environmental Genomics and Department of Microbiology and Plant Biology, University of Oklahoma, Norman, OK, 73019.

^f^State Key Joint Laboratory of Environment Simulation and Pollution Control, School of Environment, Tsinghua University, Beijing 100084, China.

^g^Department of Environmental Science, Policy and Management. University of California-Berkeley, Berkeley, CA, 94209.

*Corresponding authors:

Nicholas Bouskill, Ecology Department, Earth Sciences Division, Lawrence Berkeley National Laboratory, Berkeley, CA, 94702.

E-mail: [njbouskill@lbl.gov](mailto:njbouskill@lbl.gov)

Tel: (+1) 510-486-7490

Fax: (+1) 510-486-7152

Eoin Brodie, Ecology Department, Earth Sciences Division, Lawrence Berkeley National Laboratory, Berkeley, CA, 94720.

E-mail: elbrodie@lbl.gov

Tel: (+1) 510-486-6584

Fax: (+1) 510-486-7152

**Supplemental Tables**

Table S1: Table of physicochemical factors measured in April, 2010, 10 months following the placement of rainfall shelters.

Table S2: Multi response permutation procedure between different functional gene data sets within samples across the two different samples times across 999 permutations. The physicochemical data used in the mrpp analysis included the soil water content and water potential, the time point sampled (3 mth or 10 mths), sodium concentrations (as a proxy for solute concentration), and phosphorus concentrations. Shown below are the effect size and p-value for the treatments sampled at 3 mths and then 10 mths. The effect on community composition of time was controlled for in different analyses. Also given are the analyses between control and treatments and between treatments for the same time point (3 mth or 10 mth).

|  | **Comparison** | **Effect size** | ***p*** |
| --- | --- | --- | --- |
| Within treatments | Pre-excluded + time | 0.04 | <0.05 |
|  | Pre-excluded - time | 0.04 | <0.041 |
|  | *De-novo* + time | 0.06 | <0.008 |
|  | *De-novo* - time | 0.05 | <0.007 |
| Between treatments | Control – Pre-excl. 3 mth | 0.01 | <0.73 |
|  | Control – Pre-excl. 10 mth | 0.001 | <0.48 |
|  | Control – *De-novo* 3mth | 0.03 | <0.05 |
|  | Control – *De-novo* 10mth | 0.02 | <0.015 |
|  | Pre-excl. – *De-novo 3 mth* | 0.01 | <0.18 |
|  | Pre-excl. – *De-novo 10 mth* | 0.04 | <0.01 |

Table S3: Permutational multivariate comparisons between biological data sets and physicochemical data. (a) Total functional gene data following 3 months of throughfall exclusion. The subsequent tables are all taken from the 10 month sampling point, and represent (b) the total functional gene data, and functional gene data by category, i. Carbon cycling, ii. Nitrogen cycling, iii. Phosphorus cycling, iv. Stress genes, (v) the 4 categories analyzed together. Abbreviations; Ψ = Water potential, Na = Sodium (a proxy for solute concentration). Significant relationships (*p* < 0.05) are highlighted by bold italics.

(a)

| **Sources of variance** | **D.f** | **S.S** | **F** | **R^2^** | ***p*** |
| --- | --- | --- | --- | --- | --- |
| Treatment | 2 | 0.18 | 0.99 | 0.1 | 0.42 |
| Ψ | 1 | 0.12 | 0.85 | 0.06 | 0.64 |
| Na | 1 | 0.22 | 1.33 | 0.18 | 0.3 |
| P | 1 | 0.25 | 1.39 | 0.4 | 0.09 |
| Fe | 1 | 0.27 | 1.1 | 0.38 | 0.12 |
| Treatment x Na x P | 2 | 0.36 | 1.38 | 0.19 | 0.24 |
| Na x P | 1 | 0.46 | 1.61 | 0.07 | 0.48 |
| Na x Fe | 1 | 0.13 | 0.75 | 0.04 | 0.62 |

(b)

| **Sources of variance** | **D.f** | **S.S** | **F** | **R^2^** | ***p*** |
| --- | --- | --- | --- | --- | --- |
| Treatment | 2 | 0.35 | 1.15 | 0.16 | 0.2 |
| Ψ | 1 | 0.15 | 0.95 | 0.06 | 0.56 |
| ***Na*** | ***1*** | ***0.24*** | ***1.54*** | ***0.11*** | ***0.02*** |
| P | 1 | 0.15 | 0.9 | 0.06 | 0.54 |
| Fe | 1 | 0.15 | 0.9 | 0.06 | 0.57 |
| ***Treatment x Na x P*** | ***2*** | ***0.36*** | ***1.3*** | ***0.15*** | ***0.04*** |
| Na x P | 1 | 0.16 | 1.01 | 0.07 | 0.4 |
| Na x Fe | 1 | 0.14 | 0.86 | 0.06 | 0.7 |

1. Carbon cycling

| **Sources of variance** | **D.f** | **S.S** | **F** | **R^2^** | ***p*** |
| --- | --- | --- | --- | --- | --- |
| ***Treatment*** | ***2*** | ***5.1*** | ***1.26*** | ***0.17*** | ***0.03*** |
| Ψ | 1 | 2.4 | 1.16 | 0.08 | 0.25 |
| Na | 1 | 2.17 | 1.04 | 0.07 | 0.36 |
| P | 1 | 2.67 | 1.3 | 0.09 | 0.1 |
| Fe | 1 | 1.79 | 0.84 | 0.06 | 0.72 |
| Treatment x Na x P | 2 | 3.41 | 0.8 | 0.11 | 0.77 |
| ***Na x P*** | ***1*** | ***3.16*** | ***1.63*** | ***0.11*** | ***0.02*** |
| Na x Fe | 1 | 1.2 | 0.56 | 0.04 | 0.95 |

1. Nitrogen cycling

| **Sources of variance** | **D.f** | **S.S** | **F** | **R^2^** | ***p*** |
| --- | --- | --- | --- | --- | --- |
| ***Treatment*** | ***2*** | ***5.44*** | ***1.21*** | ***0.17*** | ***0.004*** |
| Ψ | 1 | 1.8 | 0.87 | 0.06 | 0.34 |
| Na | 1 | 2.6 | 1.14 | 0.08 | 0.17 |
| ***P*** | ***1*** | ***2.42*** | ***1.7*** | ***0.19*** | ***0.03*** |
| Fe | 1 | 1.84 | 0.78 | 0.05 | 0.85 |
| Treatment x Na x P | 2 | 3.75 | 0.81 | 0.12 | 0.78 |
| ***Na x P*** | ***1*** | ***3.3*** | ***1.57*** | ***0.11*** | ***0.008*** |
| Na x Fe | 1 | 1.5 | 0.62 | 0.04 | 0.95 |

1. Phosphorus cycling

| **Sources of variance** | **D.f** | **S.S** | **F** | **R^2^** | ***p*** |
| --- | --- | --- | --- | --- | --- |
| ***Treatment*** | ***2*** | ***4.9*** | ***1.25*** | ***0.17*** | ***0.002*** |
| Ψ | 1 | 2.4 | 1.2 | 0.08 | 0.2 |
| Na | 1 | 1.9 | 1.14 | 0.07 | 0.53 |
| ***P*** | ***1*** | ***2.67*** | ***1.4*** | ***0.11*** | ***0.04*** |
| Fe | 1 | 1.66 | 0.8 | 0.05 | 0.85 |
| Treatment x Na x P | 2 | 3.75 | 0.95 | 0.13 | 0.57 |
| ***Na x P*** | ***1*** | ***2.9*** | ***1.56*** | ***0.10*** | ***0.02*** |
| Na x Fe | 1 | 1.3 | 0.61 | 0.04 | 0.94 |

1. Stress genes

| **Sources of variance** | **D.f** | **S.S** | **F** | **R^2^** | ***p*** |
| --- | --- | --- | --- | --- | --- |
| ***Treatment*** | ***2*** | ***5.06*** | ***1.29*** | ***0.17*** | ***0.001*** |
| Ψ | 1 | 2.28 | 1.09 | 0.07 | 0.32 |
| Na | 1 | 2.23 | 1.1 | 0.07 | 0.21 |
| P | 1 | 2.62 | 1.3 | 0.09 | 0.06 |
| Fe | 1 | 1.82 | 0.88 | 0.06 | 0.68 |
| Treatment x Na x P | 2 | 3.37 | 0.85 | 0.12 | 0.75 |
| ***Na x P*** | ***1*** | ***2.76*** | ***1.44*** | ***0.09*** | ***0.02*** |
| Na x Fe | 1 | 1.33 | 0.63 | 0.04 | 0.94 |

1. 4 categories together

| **Sources of variance** | **D.f** | **S.S** | **F** | **R^2^** | ***p*** |
| --- | --- | --- | --- | --- | --- |
| Treatment | 2 | 0.37 | 1.5 | 0.16 | 0.19 |
| Ψ | 1 | 0.15 | 0.95 | 0.07 | 0.55 |
| ***Na*** | ***1*** | ***0.24*** | ***1.53*** | ***0.11*** | ***0.02*** |
| P | 1 | 0.15 | 0.9 | 0.07 | 0.55 |
| Fe | 1 | 0.15 | 0.93 | 0.07 | 0.56 |
| Treatment x Na x P | 2 | 0.35 | 1.32 | 0.15 | 0.07 |
| Na x P | 1 | 0.15 | 0.99 | 0.07 | 0.51 |
| Na x Fe | 1 | 0.14 | 0.87 | 0.06 | 0.72 |

**Supplemental Figures**

Figure S1: Weighted CCA of different aspects of the functional gene microarray data depicting the ordination of the 5 replicates per treatment after 10 months of throughfall exclusion. The different panels show (a) all functional genes, functional genes involved in (b) carbon, (c) nitrogen, (d) phosphorus, and (e) stress genes.

Figure S2: Weighted CCA of genes responsible for discriminating the treatments following short-term throughfall exclusion. Plots show genes involved in (a) carbon and (b) nitrogen cycling, (c) stress pathways and (d) phosphorus cycling. The genes used to create the ordination analysis are those that are significantly different between the soils (ANOVA, *p* < 0.05).

Figure S3: Weighted CCA of genes responsible for discriminating the sites following prolonged throughfall exclusion. (a) Oxygen stress genes, (b) genes involved in Hemicellulose breakdown, and (c) genes involved in anaerobic activity. The genes used to create the ordination analysis are those that are significantly between the different soils (ANOVA, *p* < 0.05).
